# Schemes for Hastening the Millennium

**Published:** 1899-10

**Authors:** 


### Schemes for Hastening the Millennium

Are produced with pleasing frequency in these advanced days, and it is astounding what a number of highly moral measures are born and die in the course of a single parliamentary session. This year the private members have been particularly prolific in suggestions for the purification of public manners, and the removal of that small residuum of the "Old Adam" which is still to be found in nineteenth century human nature. One is led to these observations by the introduction on Tuesday of a bill to amend the Indecent Advertisements Act of ten years ago. The promoters of the bill are Mr. Channing, Sir J. Kennaway and Mr. Samuel Smith—gentlemen who are well known in connection with various reform movements usually promoted by societies which prefix "anti" to their titles. Perhaps they are unduly sensitive, but, at any rate, these gentlemen deserve praise for having the courage to deal with what they deem to be a public scandal. The proposal in effect is to extend the Statute of 1889 to all printed or written matter offering to provide drugs, instruments or surgical or other means for the purpose of procuring, or of enabling others to procure, the miscarriage of any woman *whether she be with child or not.* The concluding words are so rich as to be well worthy of italics. The penalty for the insertion of such a notice in a newspaper is to involve the person inserting it in liability to a fine of £10 or three months' imprisonment, and the same punishment awaits the sender of indecent printed matter through the post. The courts will have to define what is or is not a print of an indecent nature.—*Pharmaceutical Journal, London.*

